# Supplementary material for: Associations of schizophrenia with arrhythmic disorders and electrocardiogram traits: a genetic exploration of population samples
Source: Br J Psychiatry. Author manuscript; Available in PMC 2024 Dec 5. (PMC7616879; doi:10.1192/bjp.2024.165)
Supplement: Supplementary Information [file EMS198191-supplement-Supplementary_Information.pdf]

***Supplement to:* Associations of schizophrenia with arrhythmic disorders and electrocardiogram traits: a genetic exploration of population samples**

Jorien L Treur, Anaïs B Thijssen, Dirk JA Smit, Rafik Tadros, Rada R Veeneman, Damiaan Denys, Jentien M Vermeulen, Julien Barc, Jacob Bergstedt, Joëlle A Pasman, Connie R Bezzina, Karin J H Verweij

1. Local genetic correlation analyses

a. Supplementary Figure S1 (overlap in locally correlating regions across trait-pairs) 2

b. Supplementary Figures S2 to 10 (Manhattan plots with locally correlating regions) 3-7

c. Supplementary Table S1 (global and MAF-stratified genetic correlations) 8

d. Supplementary Table S2 (list of locally correlating regions) 9-11

2. Functional annotation analyses

a. Supplementary Figure S11 (GWAS catalogue phenotypes) 12

b. Supplementary Figure S12 & S13 (GO biological processes enrichment) 13-14

c. Supplementary Figure S14 (Differential tissue expression) 15

3. Mendelian randomization analyses

a. Supplementary Figures S15 to 16 (scatter plots causal findings) 16

b. Supplementary Figures S17 to 18 (funnel plots causal findings) 17

c. Supplementary Figures S19 to 20 (leave-one-out analyses causal findings) 18-19

d. Supplementary Table S3 (Cochran's Q statistic to assess heterogeneity) 20

e. Supplementary Table S4 (Multivariable MR analyses) 21

1. Local genetic correlation analyses

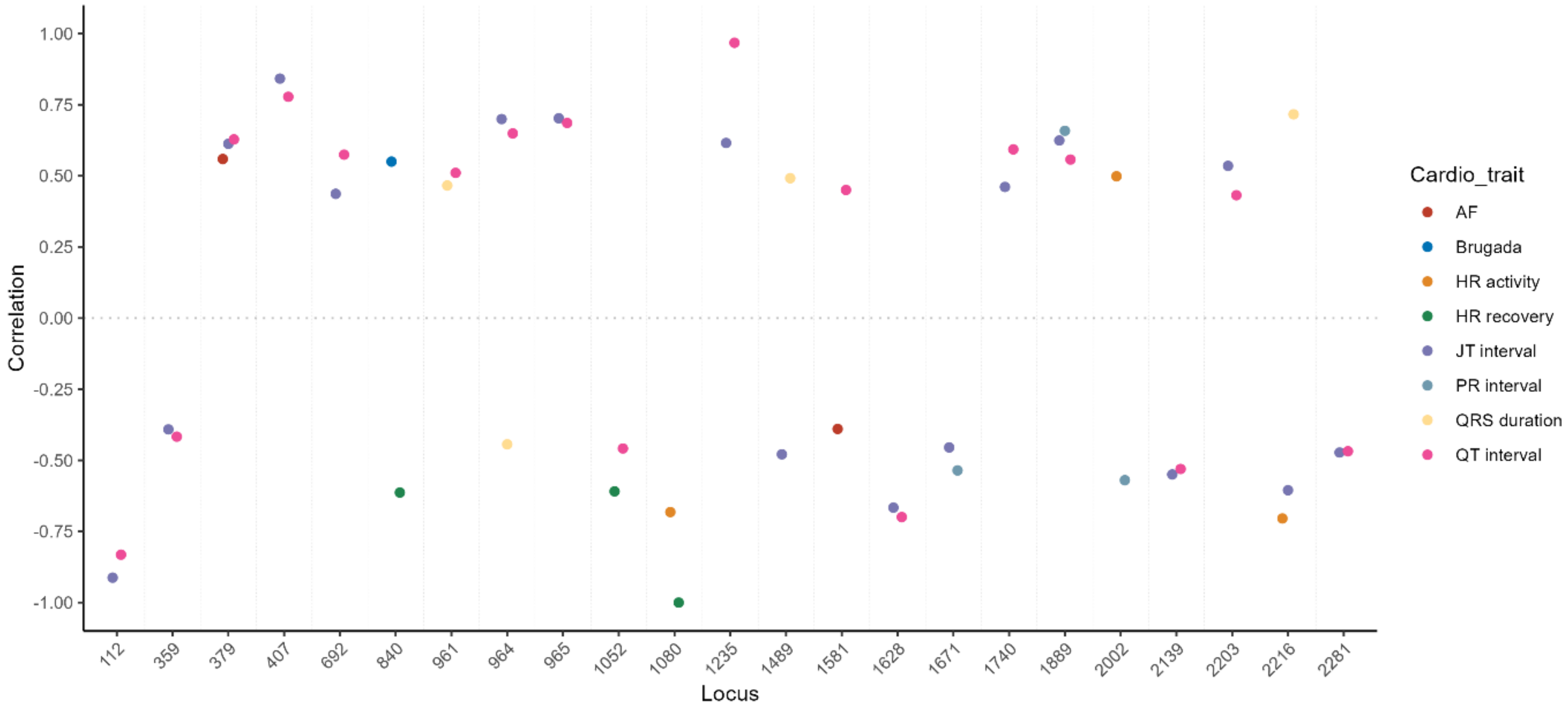

Figure S1. Regions that emerged in two or more cardio trait – schizophrenia local correlation analyses are plotted. The x-axis shows the 23 regions that overlapped between LAVA analyses and the y-axis shows the local genetic correlation between the cardio traits and schizophrenia for the involved regions.

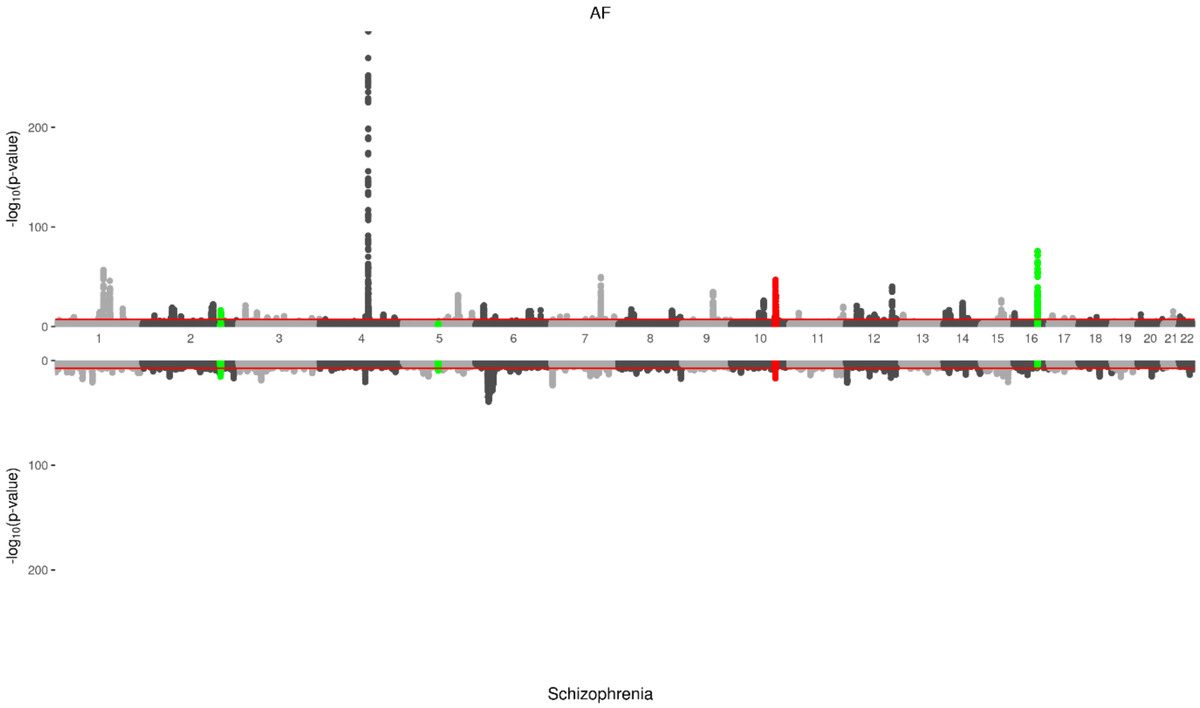

Figure S2. Miami plots for the Atrial fibrillation and schizophrenia GWASs. SNPs from LAVA regions that showed significant (FDR-corrected) local genetic correlations are shown in red (correlation was negative) and green (correlation was positive).

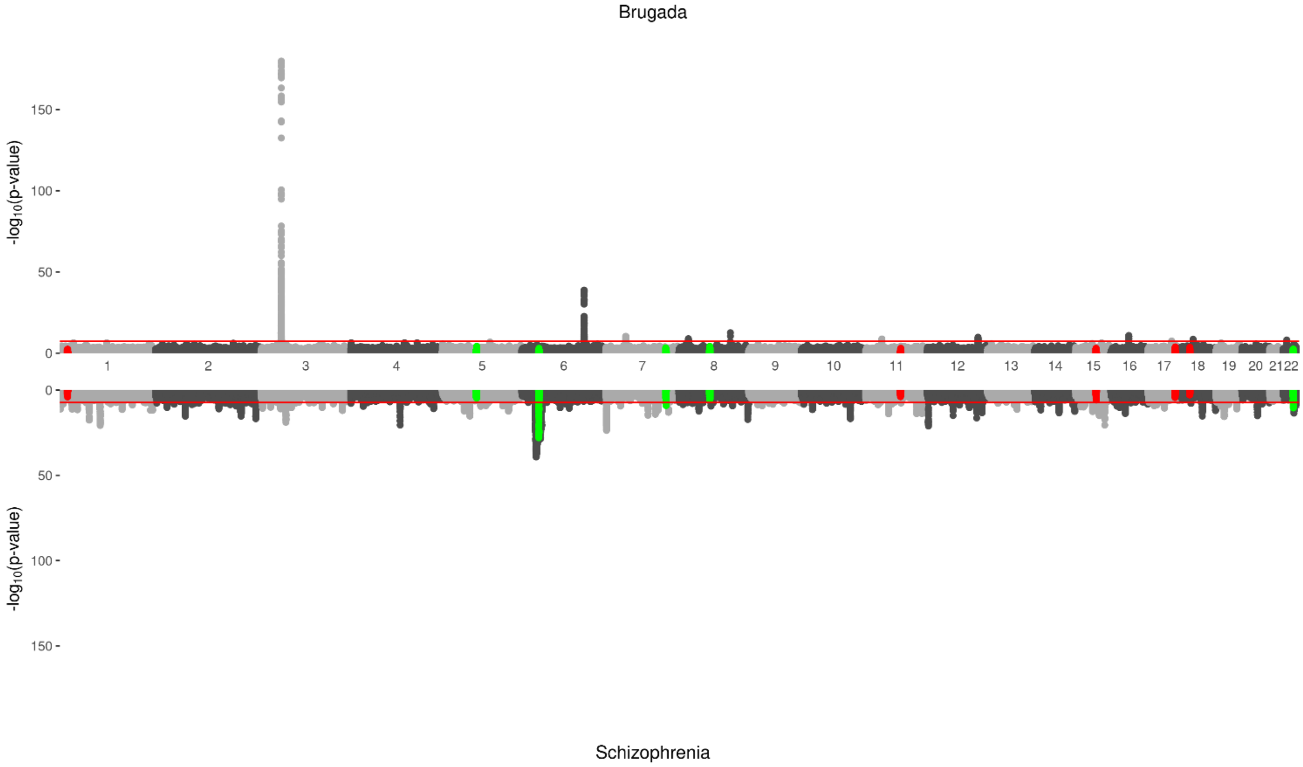

Figure S3. Miami plots for the Brugada syndrome and schizophrenia GWASs. SNPs from LAVA regions that showed significant (FDR-corrected) local genetic correlations are shown in red (correlation was negative) and green (correlation was positive).

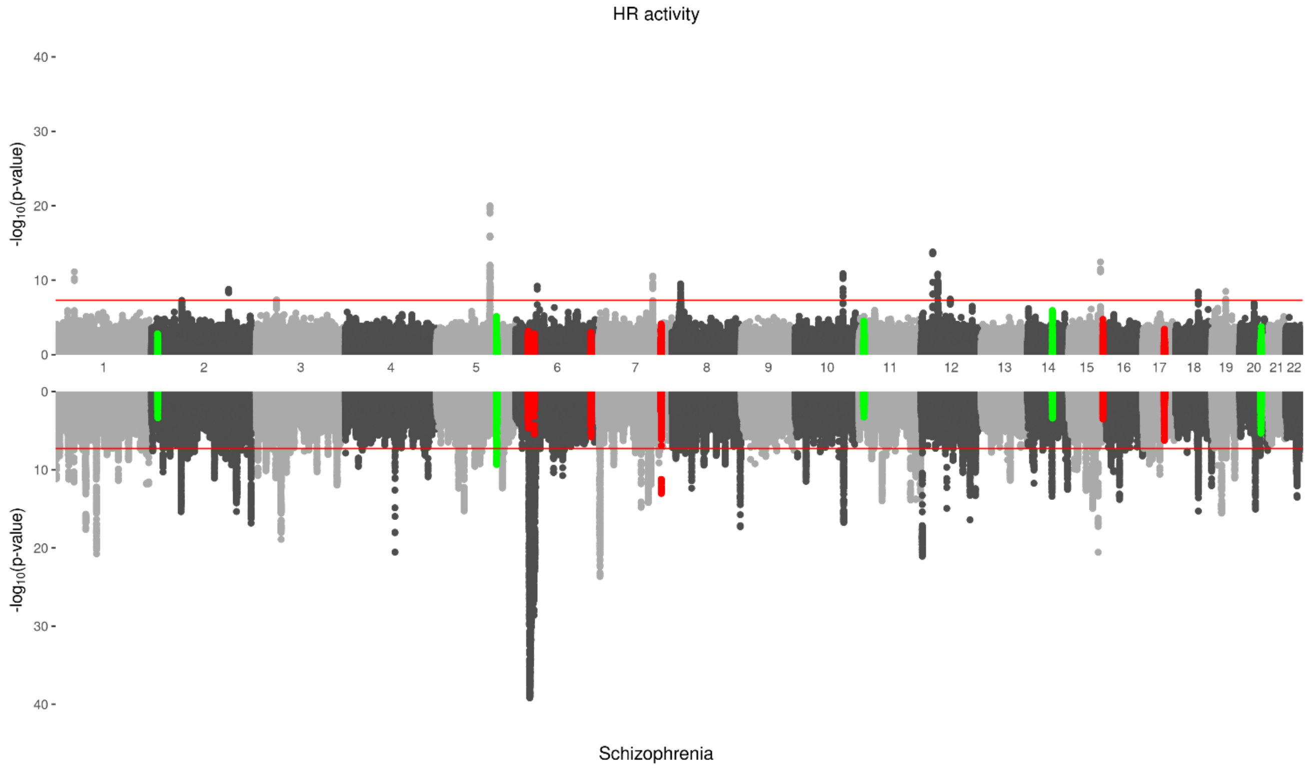

Figure S4. Miami plots for the heart rate during activity and schizophrenia GWASs. SNPs from LAVA regions that showed significant (FDR-corrected) local genetic correlations are shown in red (correlation was negative) and green (correlation was positive).

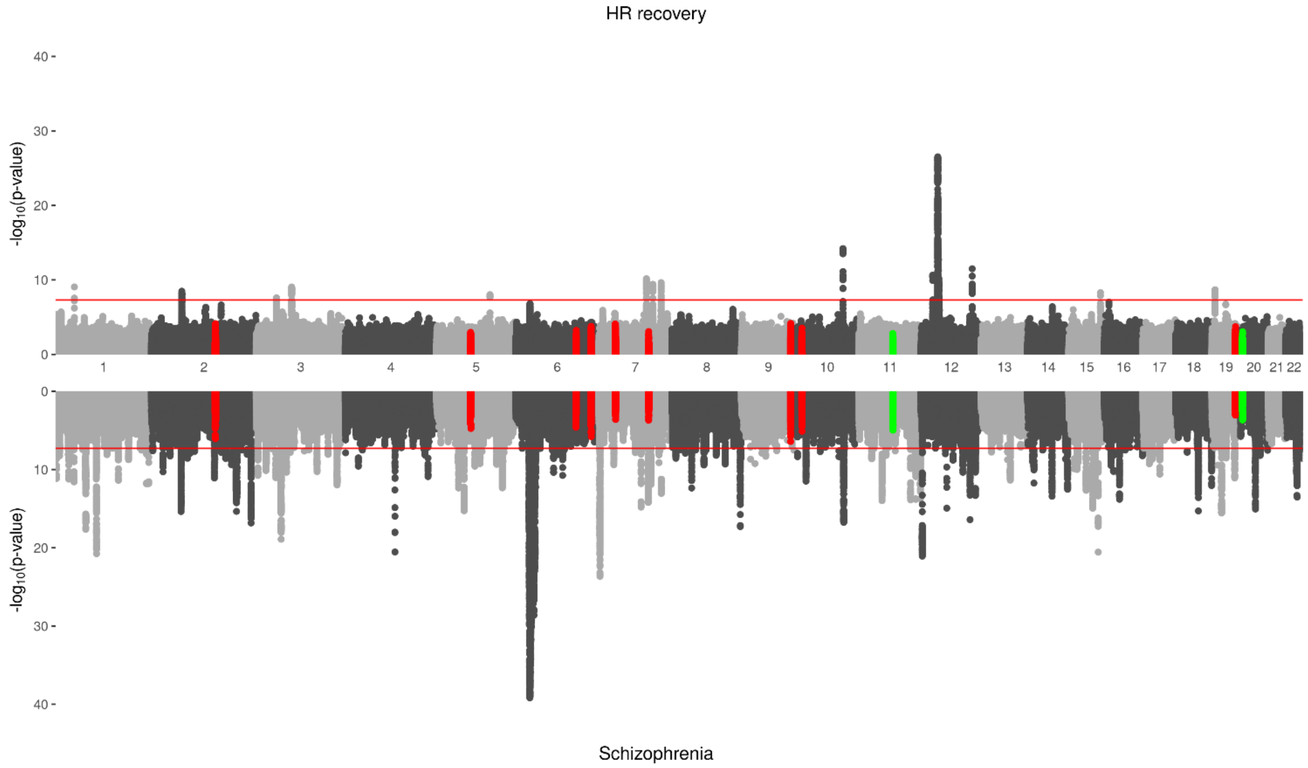

Figure S5. Miami plots for the heart rate during recovery and schizophrenia GWASs. SNPs from LAVA regions that showed significant (FDR-corrected) local genetic correlations are shown in red (correlation was negative) and green (correlation was positive).

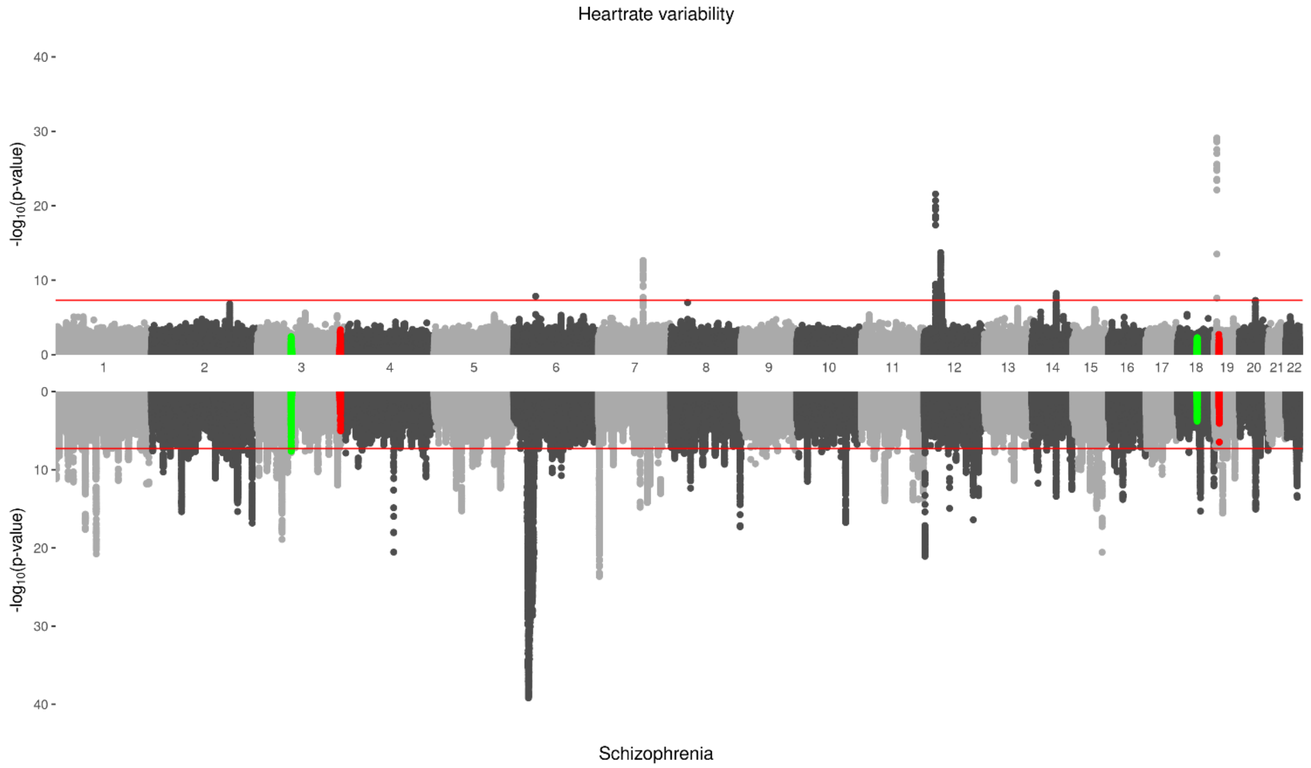

Figure S6. Miami plots for the heart rate variability and schizophrenia GWASs. SNPs from LAVA regions that showed significant (FDR-corrected) local genetic correlations are shown in red (correlation was negative) and green (correlation was positive).

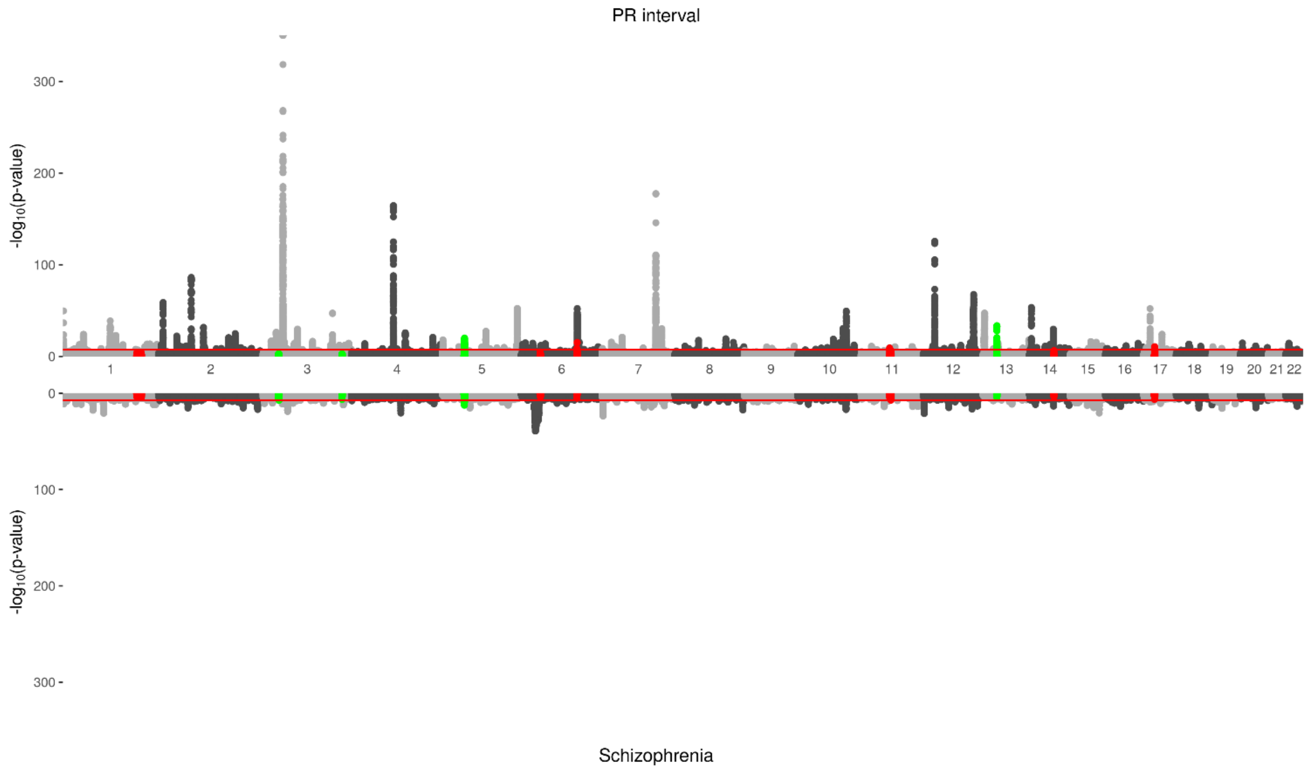

Figure S7. Miami plots for the PR interval and schizophrenia GWASs. SNPs from LAVA regions that showed significant (FDR-corrected) local genetic correlations are shown in red (correlation was negative) and green (correlation was positive).

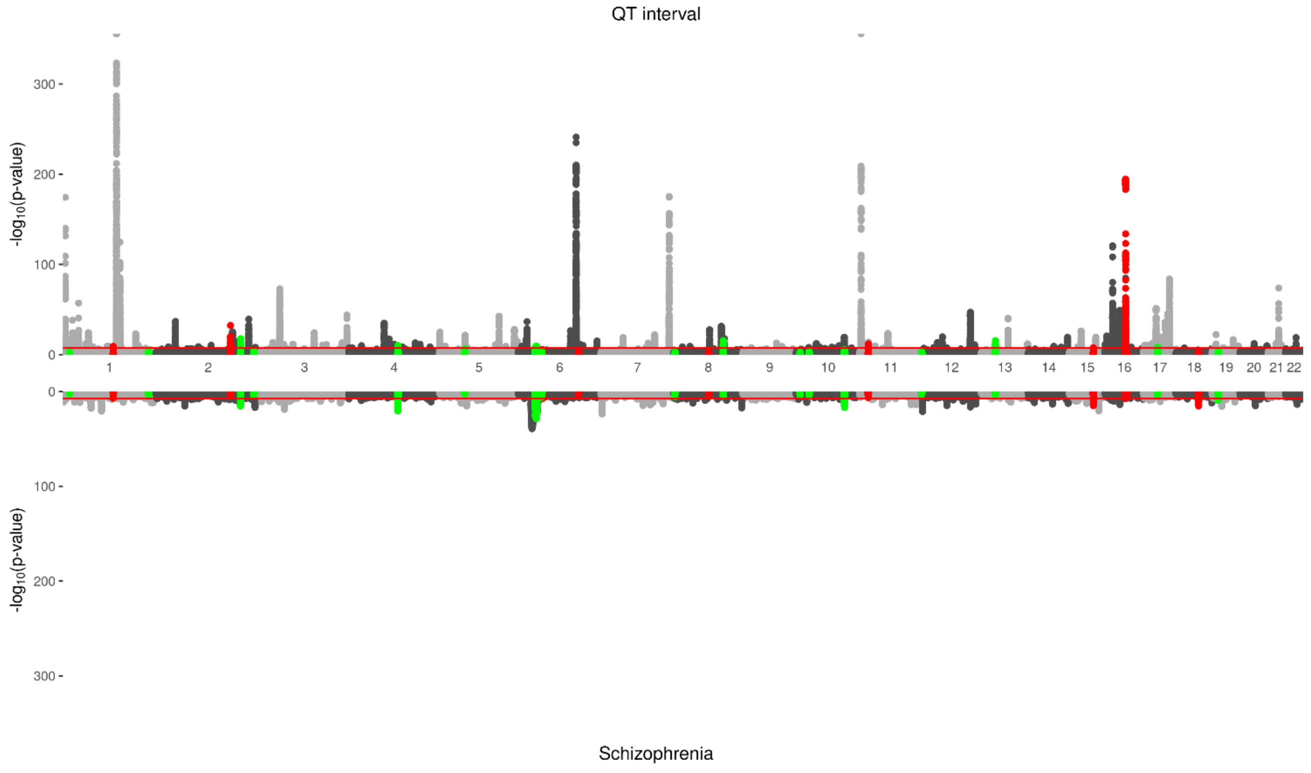

Figure S8. Miami plots for the QT interval and schizophrenia GWASs. SNPs from LAVA regions that showed significant (FDR-corrected) local genetic correlations are shown in red (correlation was negative) and green (correlation was positive).

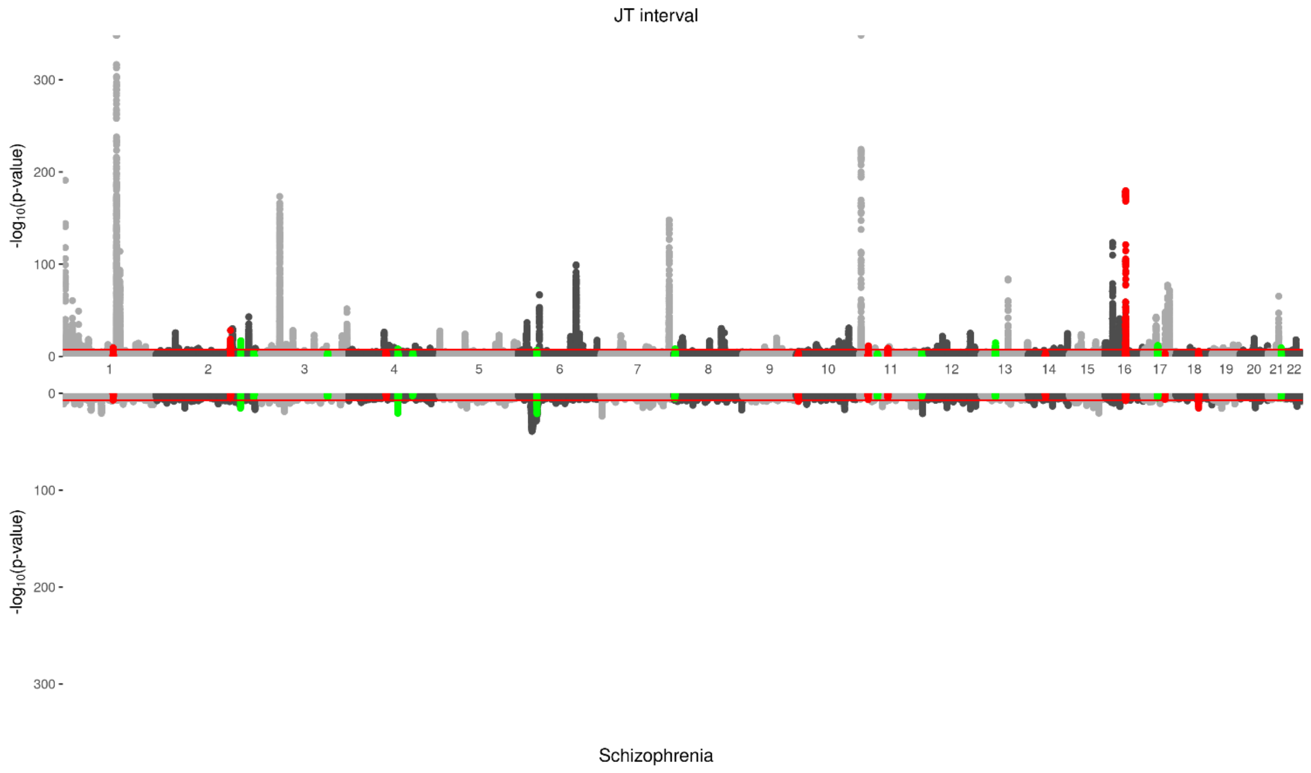

Figure S9. Miami plots for the JT interval and schizophrenia GWASs. SNPs from LAVA regions that showed significant (FDR-corrected) local genetic correlations are shown in red (correlation was negative) and green (correlation was positive).

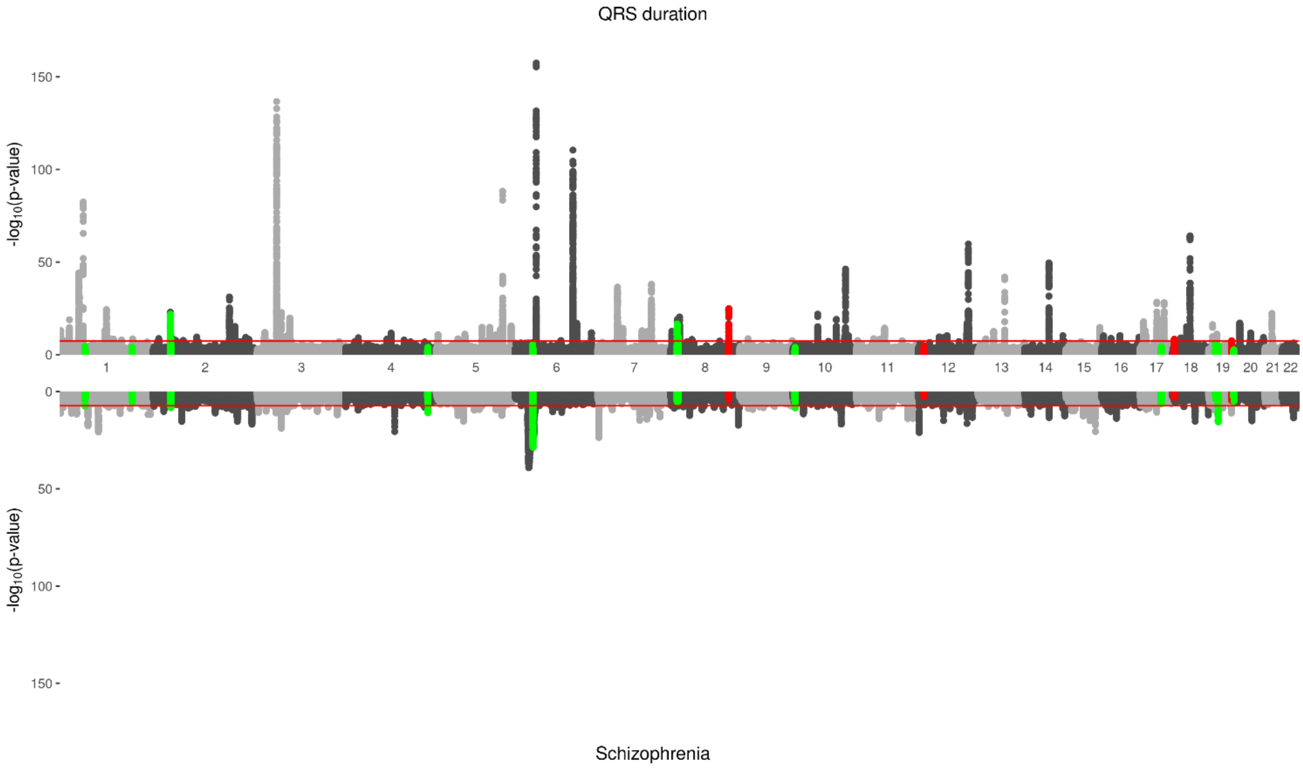

Figure S10. Miami plots for the QRS interval and schizophrenia GWASs. SNPs from LAVA regions that showed significant (FDR-corrected) local genetic correlations are shown in red (correlation was negative) and green (correlation was positive).

Table S1. Global and MAF-stratified genetic correlations of schizophrenia with all cardiac traits

**Cardiac traits**	**Global**		***MAF 0·05-0·11***		**MAF 0·11-0·22**		**MAF 0·22-0·36**		**MAF 0·36-0·50**	
	**r_g (95% CIs)**	***p-value***	**r_G (95% CIs)**	***p-value***	**r_G (95% CIs)**	***p-value***	**r_G (95% CIs)**	***p-value***	**r_G (95% CIs)**	***p-value***
AF	-0·034 (-0·075 to -0·007)	0·106	-0·073 (-0·165 to 0·020)	0·125	-0·017 (-0·085 to 0·051)	0·621	-0·027 (-0·087 to 0·034)	0·388	-0·021 (-0·085 to 0·043)	0·523
Brugada	0·137 (0·061 to 0·213)	4E-04	0·153 (-0·099 to 0·404)	0·234	0·217 (0·048 to 0·385)	0·012	0·163 (0·052 to 0·273)	0·004	0·085 (-0·018 to 0·188)	0·105
HR act	0·012 (-0·052 to 0·076)	0·718	-0·007 (-0·159 to 0·145)	0·926	-0·017 (-0·114 to 0·080)	0·735	-0·007 (-0·101 to 0·088)	0·891	0·026 (-0·054 to 0·107)	0·523
HR rec	-0·072 (-0·142 to -0·002)	0·044	-0·128 (-0·299 to0·044)	0·144	-0·140 (-0·260 to-0·020)	0·022	-0·070 (-0·170 to 0·030)	0·167	-0·055 (-0·142 to 0·033)	0·218
HR var	-0·006 (-0·098 to0·085)	0·895	0·148 (-0·068 to 0·364)	0·178	-0·035 (-0·177 to 0·107)	0·628	-0·062 (-0·197 to 0·073)	0·367	-0·027 (-0·125 to 0·071)	0·588
PR	-0·023 (-0·061 to 0·015)	0·242	-0·036 (-0·120 to 0·048)	0·399	-0·006 (-0·068 to 0·056)	0·847	-0·002 (-0·053 to0·049)	0·931	-0·050 (-0·115 to 0·015)	0·130
QT	0·019 (-0·020 to 0·058)	0·343	-0·031 (-0·109 to 0·048)	0·442	0·026 (-0·043 to 0·095)	0·463	0·041 (-0·015 to 0·098)	0·153	0·027 (-0·026 to 0·081)	0·316
JT	0·010 (-0·029 to 0·048)	0·618	-0·020 (-0·088 to 0·049)	0·573	0·001 (-0·073 to 0·075)	0·978	0·014 (-0·040 to 0·067)	0·618	0·031 (-0·025 to 0·087)	0·279
QRS	0·021 (-0·024 to 0·067)	0·360	0·009 (-0·118 to 0·136)	0·894	0·041 (-0·027 to 0·109)	0·233	0·043 (-0·019 to 0·104)	0·174	0·002 (-0·063 to 0·068)	0·949

AF=Atrial Fibrillation, HR = Heart Rate, MAF=minor allele frequency, r_G=genetic correlation

Table S2. List of regions showing local correlation (FDR-corrected)

Region number	Chromosome	Start location	Stop location	n SNPs	Cardiac trait	r_g	*p*-value	*p*-value FDR
379	2	199654970	201324132	2558	AF	0.313	0.000	0.000
849	5	87943483	89584466	2073	AF	0.238	0.001	0.046
1581	10	104206838	106142283	3573	AF	0.152	0.000	0.000
2150	16	72089512	73140781	1760	AF	0.205	0.000	0.002
20	1	18427821	19238924	2377	Brugada	0.676	0.000	0.004
840	5	77290256	79005158	3231	Brugada	0.303	0.000	0.025
957	6	30715007	31106493	2359	Brugada	0.328	0.000	0.025
1209	7	130418705	131856481	3251	Brugada	0.282	0.001	0.030
1294	8	57436723	58675117	3348	Brugada	0.323	0.001	0.042
1686	11	79802287	80726012	2292	Brugada	0.340	0.002	0.044
2072	15	69089816	70767983	3252	Brugada	0.309	0.001	0.026
2228	17	71466954	72383472	2383	Brugada	0.207	0.000	0.025
2258	18	24088164	25305260	2670	Brugada	0.318	0.002	0.044
2483	22	38718590	40378783	2744	Brugada	0.200	0.001	0.042
216	2	11230350	11995765	1982	HR act	0.378	0.003	0.041
891	5	136949854	139408116	3133	HR act	0.270	0.001	0.016
948	6	23939307	24950379	2910	HR act	0.263	0.001	0.016
963	6	32454578	32539567	35	HR act	0.670	0.000	0.002
1080	6	156762741	158220143	2971	HR act	0.465	0.000	0.007
1213	7	136113468	137657459	2911	HR act	0.317	0.000	0.009
1620	11	7915072	9080939	3095	HR act	0.687	0.000	0.007
2002	14	72665320	73373373	1880	HR act	0.249	0.002	0.040
2099	15	99925973	100742117	2538	HR act	0.471	0.001	0.013
2216	17	54950108	56245227	2868	HR act	0.496	0.000	0.009
2413	20	49236419	50653620	3818	HR act	0.384	0.000	0.007
337	2	147252197	149568875	3739	HR rec	0.315	0.000	0.013
840	5	77290256	79005158	3333	HR rec	0.376	0.001	0.013
1052	6	122929291	123854857	2184	HR rec	0.371	0.002	0.020
1080	6	156762741	158220143	2971	HR rec	1.000	0.000	0.000
1126	7	27351287	28890886	3541	HR rec	0.430	0.001	0.019
1191	7	105312564	106399739	2733	HR rec	0.327	0.004	0.043
1474	9	130902728	132193798	2025	HR rec	0.256	0.002	0.020
1498	10	10969482	11856924	2327	HR rec	0.312	0.001	0.019
1682	11	75445254	76518906	2238	HR rec	0.570	0.000	0.013
2358	19	51259179	51903804	1876	HR rec	0.256	0.005	0.046
2374	20	4591848	5379450	2393	HR rec	0.409	0.001	0.019
112	1	153410810	154685545	2004	JT	0.833	0.000	0.000
359	2	174118390	174927563	1971	JT	0.153	0.002	0.025
379	2	199654970	201324132	2557	JT	0.375	0.000	0.000
407	2	230541093	231847386	3217	JT	0.709	0.000	0.002
554	3	151904022	153406718	3237	JT	0.418	0.001	0.015
670	4	76497359	78045637	3781	JT	0.242	0.004	0.046
692	4	102544804	104384534	3450	JT	0.191	0.002	0.028
724	4	135809543	137400031	3574	JT	0.409	0.001	0.022
964	6	32539568	32586784	495	JT	0.489	0.000	0.000

965	6	32586785	32629239	678	JT	0.493	0.000	0.000
1235	8	1628040	2070902	1519	JT	0.379	0.001	0.015
1489	10	3225337	3880322	1843	JT	0.229	0.002	0.029
1628	11	16383387	17583948	2383	JT	0.444	0.000	0.001
1648	11	35500368	36331189	1934	JT	0.361	0.003	0.039
1671	11	60515106	61717117	1975	JT	0.207	0.003	0.036
1740	12	60317	1078397	1366	JT	0.213	0.004	0.045
1889	13	46493237	47433528	2042	JT	0.390	0.000	0.010
1988	14	56206431	57460781	3319	JT	0.164	0.003	0.033
2139	16	58508979	59885499	3527	JT	0.302	0.000	0.000
2203	17	37361179	38880481	2387	JT	0.286	0.000	0.003
2216	17	54950108	56245227	2880	JT	0.366	0.002	0.025
2281	18	52512524	53762996	2161	JT	0.223	0.000	0.010
2454	21	40480832	41344742	2645	JT	0.341	0.001	0.015
156	1	202583885	204092537	3276	PR	0.374	0.001	0.027
163	1	212347583	213958292	3631	PR	0.619	0.000	0.012
449	3	28649811	29877022	3633	PR	0.267	0.002	0.045
577	3	176931164	178110322	2503	PR	0.255	0.002	0.046
814	5	44049879	45305566	1873	PR	0.587	0.000	0.002
815	5	45305567	46388848	1784	PR	0.457	0.000	0.011
971	6	34979271	36346353	2611	PR	0.275	0.001	0.025
1046	6	117674077	118529071	1882	PR	0.253	0.001	0.025
1671	11	60515106	61717117	1963	PR	0.287	0.001	0.025
1674	11	64594823	66782661	3031	PR	0.289	0.002	0.043
1889	13	46493237	47433528	2027	PR	0.433	0.000	0.001
2002	14	72665320	73373373	1884	PR	0.325	0.001	0.026
2196	17	27344402	29783141	3036	PR	0.382	0.002	0.045
56	1	65894185	66778015	1739	QRS	0.265	0.002	0.031
157	1	204092538	205009623	2072	QRS	0.321	0.001	0.018
241	2	36867390	37603360	1988	QRS	0.644	0.000	0.001
758	4	175959698	177129678	2637	QRS	0.189	0.002	0.026
961	6	31427210	32208901	2274	QRS	0.217	0.000	0.012
964	6	32539568	32586784	495	QRS	0.197	0.000	0.008
1246	8	8064601	8589770	1573	QRS	0.209	0.002	0.031
1248	8	9167796	9835863	1795	QRS	0.338	0.004	0.050
1350	8	124001020	125453322	3282	QRS	0.397	0.000	0.000
1489	10	3225337	3880322	1843	QRS	0.242	0.002	0.026
1753	12	12721875	13559527	1700	QRS	0.291	0.001	0.018
2216	17	54950108	56245227	2880	QRS	0.513	0.000	0.008
2241	18	2839843	3722828	2355	QRS	0.254	0.001	0.015
2321	19	13968320	14891100	1988	QRS	0.488	0.000	0.004
2327	19	18504868	19749166	2171	QRS	0.213	0.001	0.020
2357	19	50451926	51259178	1824	QRS	0.262	0.000	0.009
2362	19	54042241	54602370	1760	QRS	0.423	0.001	0.018
18	1	16732169	17557746	1055	QT	0.407	0.003	0.031
112	1	153410810	154685545	2004	QT	0.692	0.000	0.001
184	1	234365797	235097113	2056	QT	0.247	0.006	0.045
359	2	174118390	174927563	1971	QT	0.174	0.001	0.009

379	2	199654970	201324132	2557	QT	0.395	0.000	0.000
407	2	230541093	231847386	3217	QT	0.605	0.000	0.002
692	4	102544804	104384534	3450	QT	0.330	0.000	0.002
823	5	55968967	56896890	2065	QT	0.311	0.003	0.029
958	6	31106494	31250556	1728	QT	0.332	0.001	0.008
959	6	31250557	31320268	1337	QT	0.735	0.000	0.000
960	6	31320269	31427209	1554	QT	0.375	0.000	0.001
961	6	31427210	32208901	2274	QT	0.261	0.000	0.001
964	6	32539568	32586784	495	QT	0.421	0.000	0.000
965	6	32586785	32629239	678	QT	0.471	0.000	0.000
966	6	32629240	32682213	776	QT	0.297	0.000	0.001
976	6	40778402	42103738	3117	QT	0.230	0.002	0.022
977	6	42103739	43770626	3153	QT	0.246	0.001	0.010
1052	6	122929291	123854857	2185	QT	0.210	0.003	0.031
1235	8	1628040	2070902	1519	QT	0.937	0.000	0.000
1304	8	68683824	70004665	3177	QT	0.664	0.000	0.001
1332	8	103716054	104467845	1953	QT	0.226	0.004	0.036
1493	10	6234719	6897037	1754	QT	0.177	0.006	0.045
1511	10	23365686	24489447	2150	QT	0.470	0.002	0.021
1581	10	104206838	106142283	3571	QT	0.203	0.001	0.018
1628	11	16383387	17583948	2383	QT	0.489	0.000	0.000
1740	12	60317	1078397	1366	QT	0.352	0.000	0.008
1889	13	46493237	47433528	2042	QT	0.311	0.001	0.011
2079	15	78514102	79292536	1832	QT	0.611	0.000	0.000
2139	16	58508979	59885499	3527	QT	0.281	0.000	0.000
2141	16	61092845	62607090	2690	QT	0.327	0.002	0.022
2203	17	37361179	38880481	2387	QT	0.187	0.004	0.034
2281	18	52512524	53762996	2161	QT	0.219	0.002	0.021
2319	19	11681979	13213186	2745	QT	0.221	0.005	0.045
483	3	71223282	72334704	981	HRV	0.456	0.000	0.001
584	3	186602046	187939199	1305	HRV	0.296	0.001	0.045
2274	18	45718493	46558306	735	HRV	0.467	0.001	0.045
2318	19	10028841	11681978	672	HRV	0.540	0.000	0.001

2. Functional annotation analyses

A

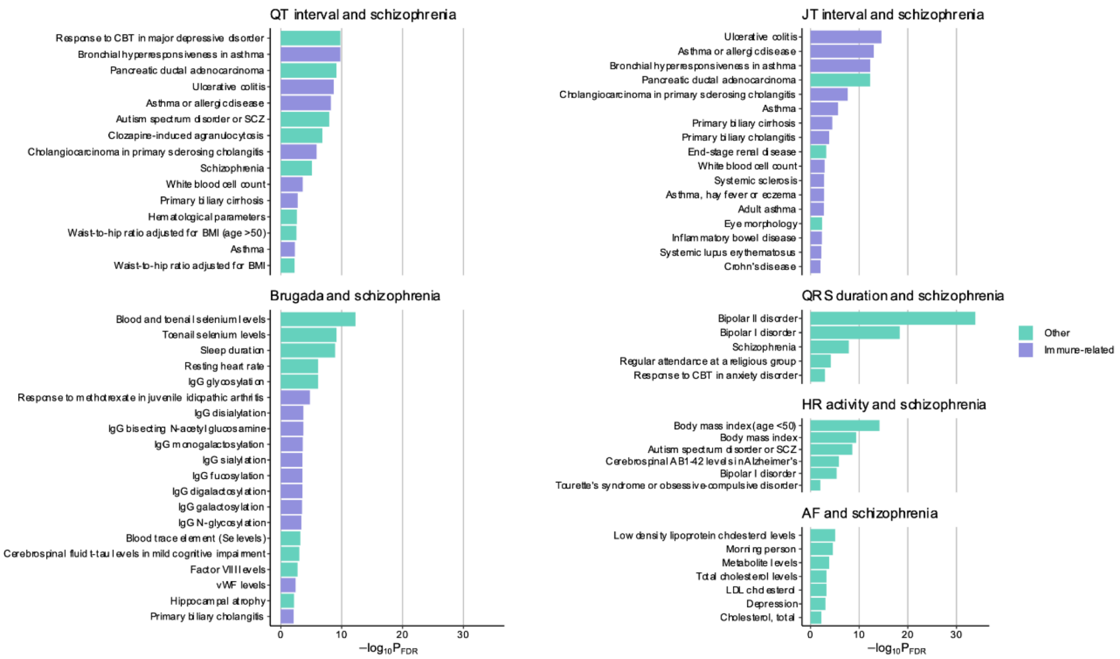

B

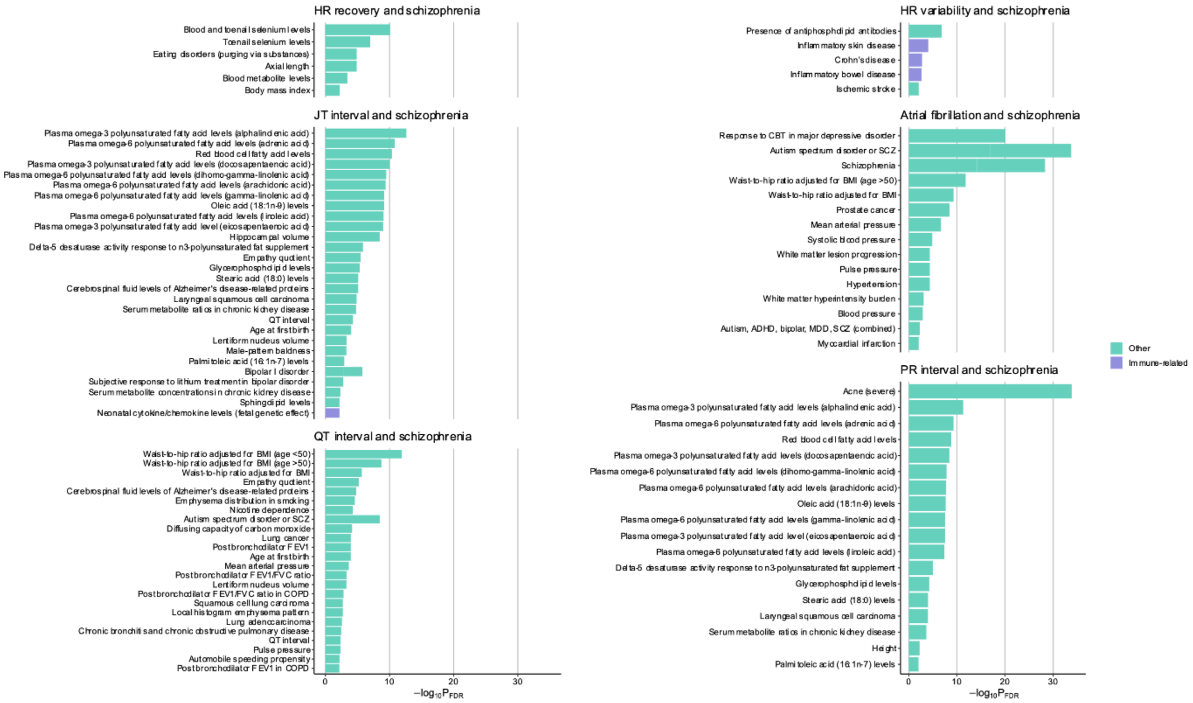

Figure S11. Previously reported trait associations from GWAS catalogue for genes in regions with a significant SCZ-cardiac trait correlation (excluding HLA region). **Panel A)** shows results for genes in regions with a positive SCZ-cardiac trait correlation; **panel B)** shows results for genes in regions with a negative correlation.

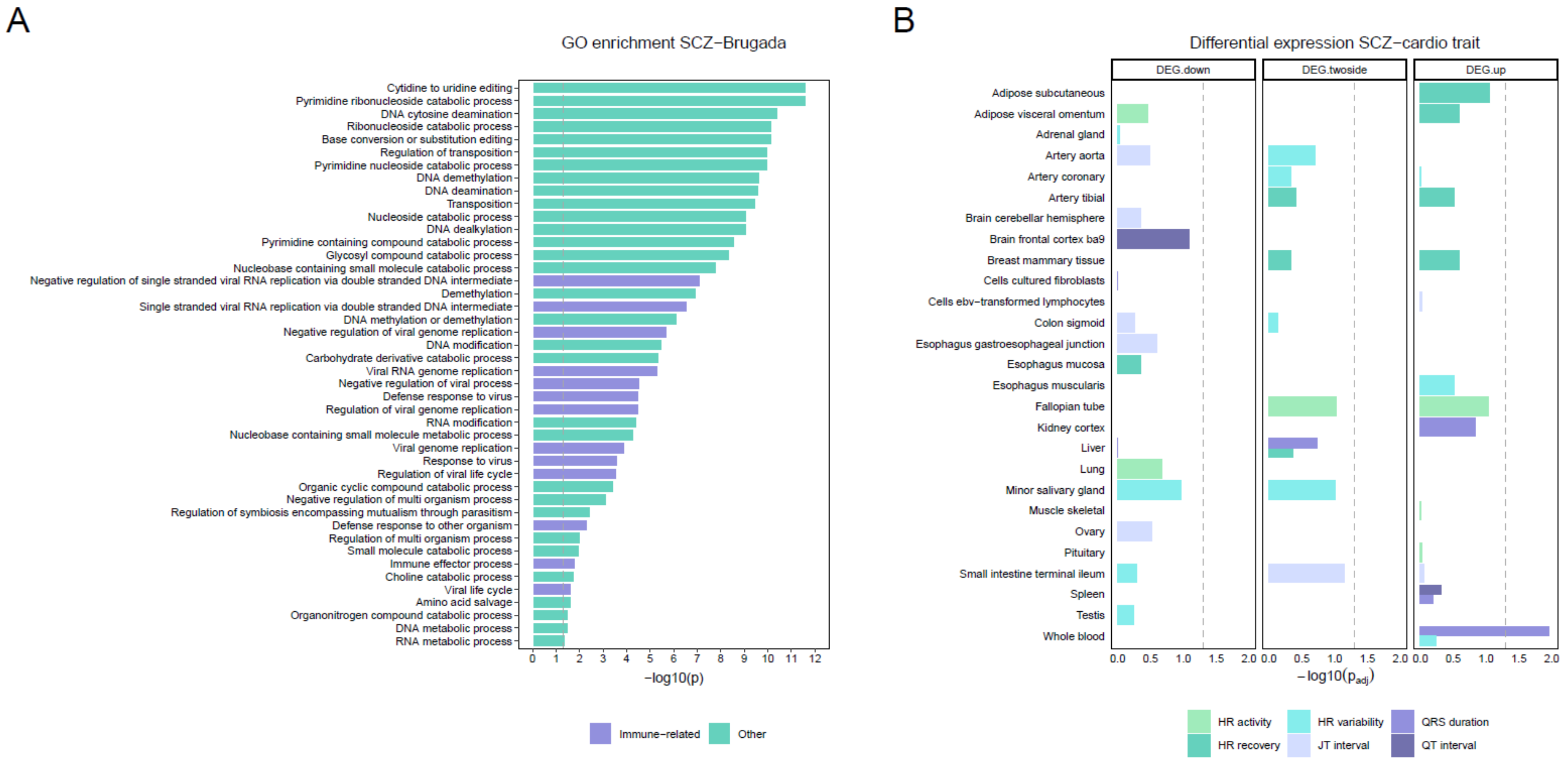

Figure S12. Functional significance of genes in regions that correlate positively between schizophrenia and an arrhythmic disorder or ECG trait. **Panel A** shows significant enrichment (after FDR-correction) in biological process GO terms for all genes in regions with a positive genetic correlation between schizophrenia and Brugada. GO terms that are primarily related to viral response and immune processes are highlighted in purple. **Panel B** shows differential tissue expression (downregulation, upregulation, and combined, at marginal $p<.05$) for genes in regions that showed a positive genetic correlation between a schizophrenia and a cardiac trait pair. The x-axes in both panels show the log-transformed p-value after FDR-correction, with the dashed lines indicating the significance threshold ($p_{FDR}<.05$).

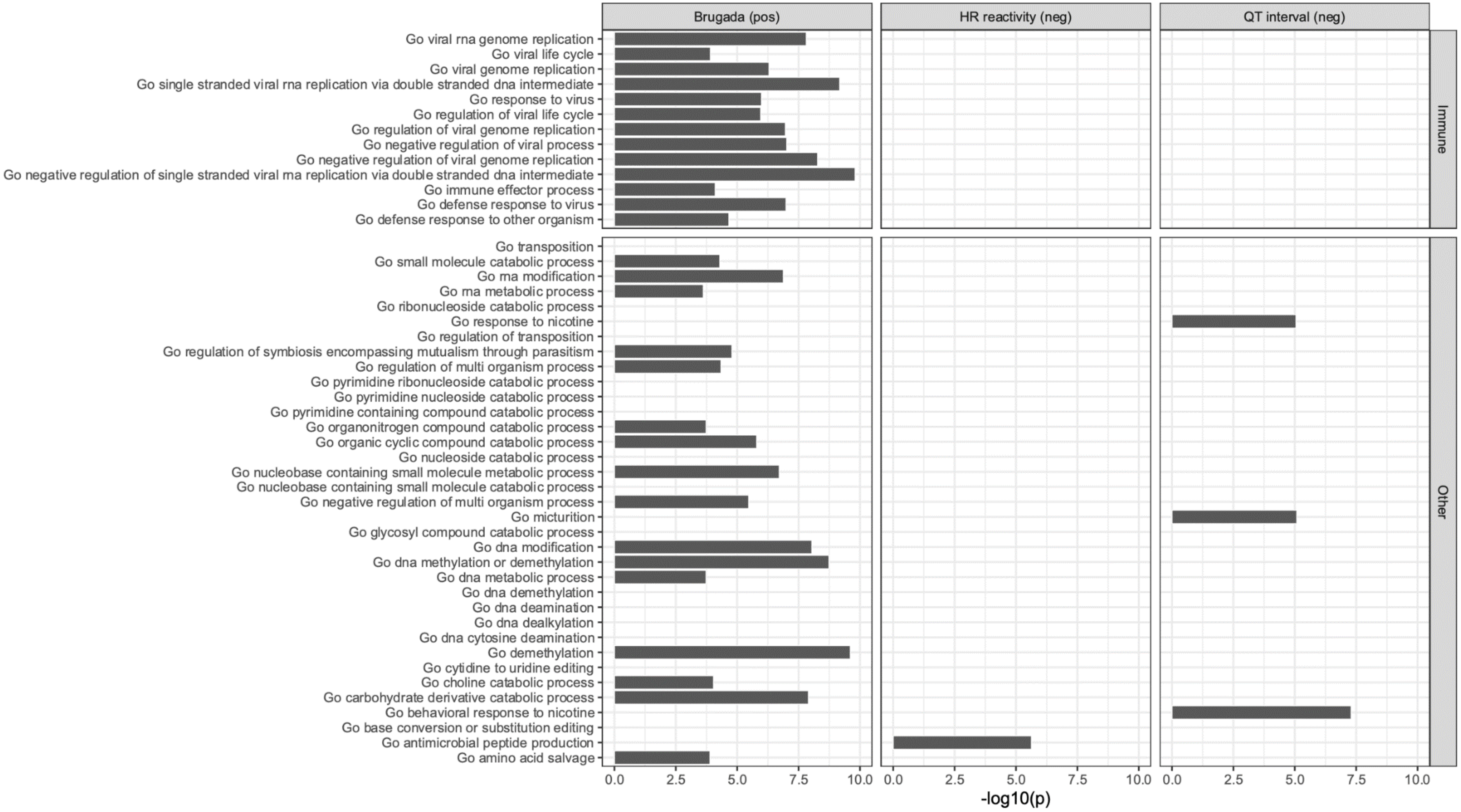

Figure S13 Full results of GO biological processes enrichment for genes in regions with a significant SCZ-cardiac trait association (excluding HLA region). Only results for trait pairs with any significant enrichment are plotted. Results for processes associated with immune function or viral response (from different GO branches) are shown at the top.

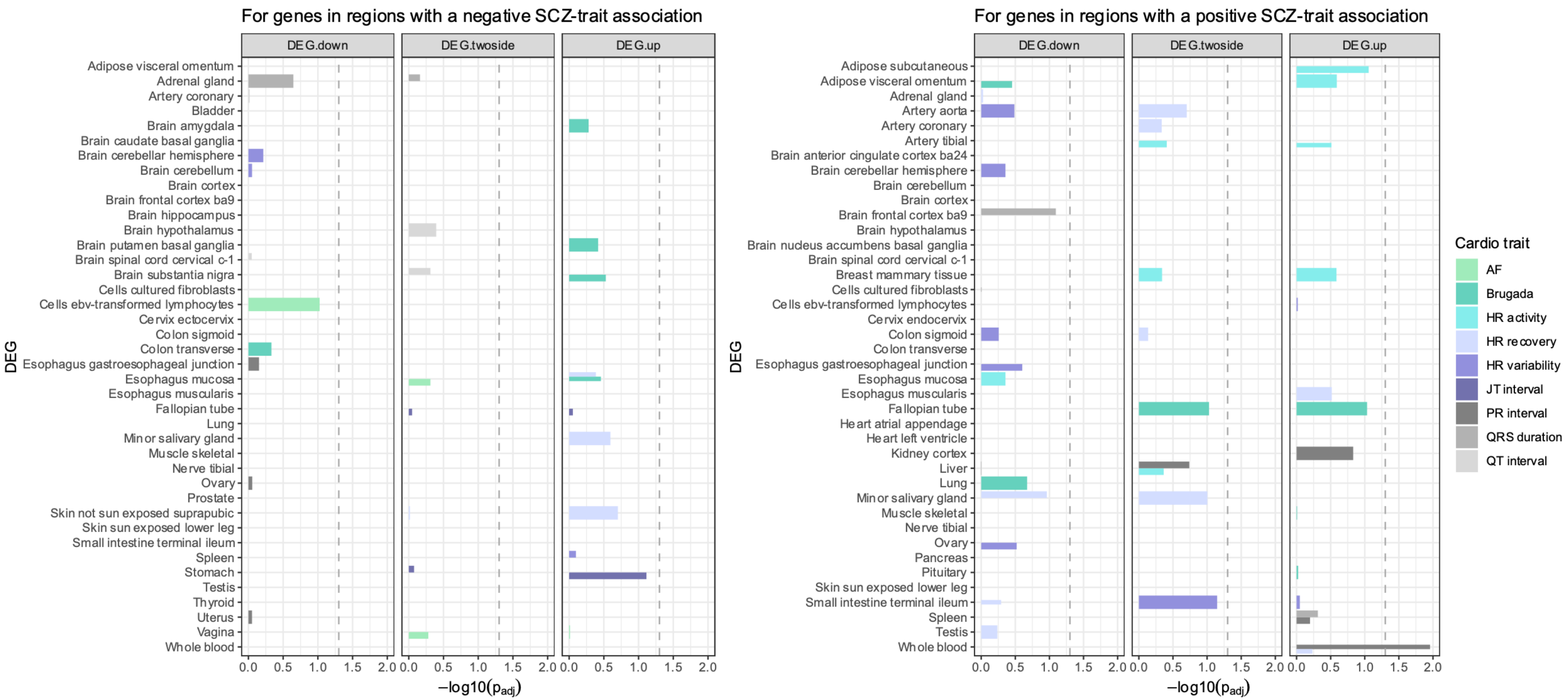

Figure S14. Full results for differential tissue expression across the 30 available tissue types of the GTEx project for genes in regions with a significant SCZ-cardiac trait association (excluding HLA region), with the left panel showing results for genes in negatively associated regions, and the right panel for genes in positively associated reg

3. Mendelian randomization analyses

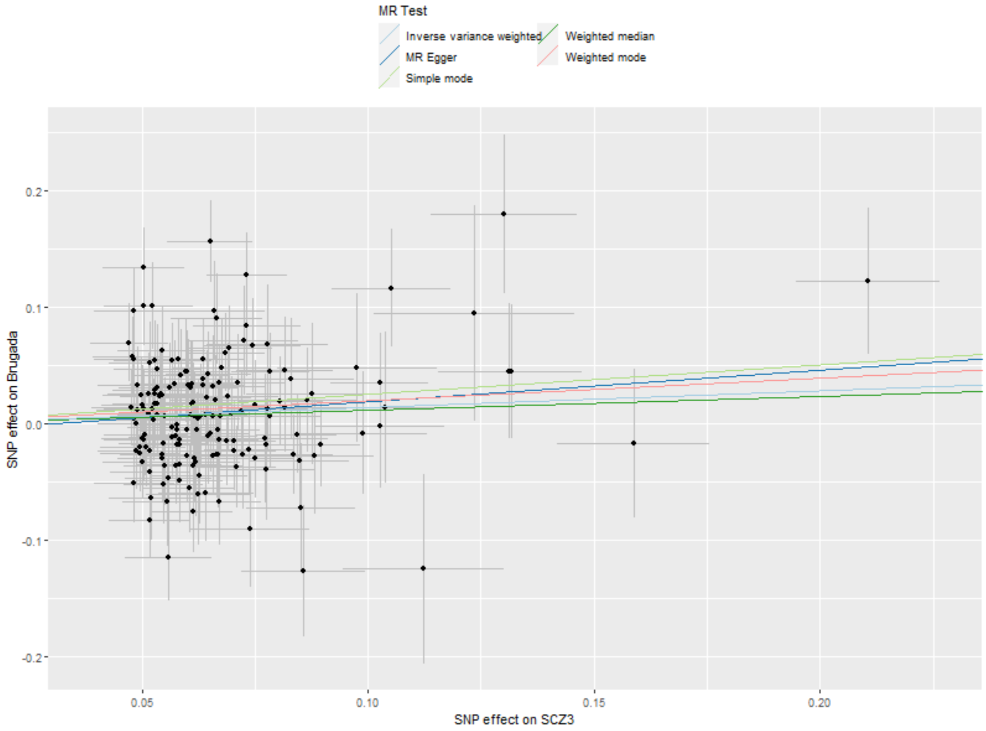

Figure S14. Scatter plot for the Mendelian randomization results with liability to schizophrenia as the exposure and Brugada syndrome as the outcome.

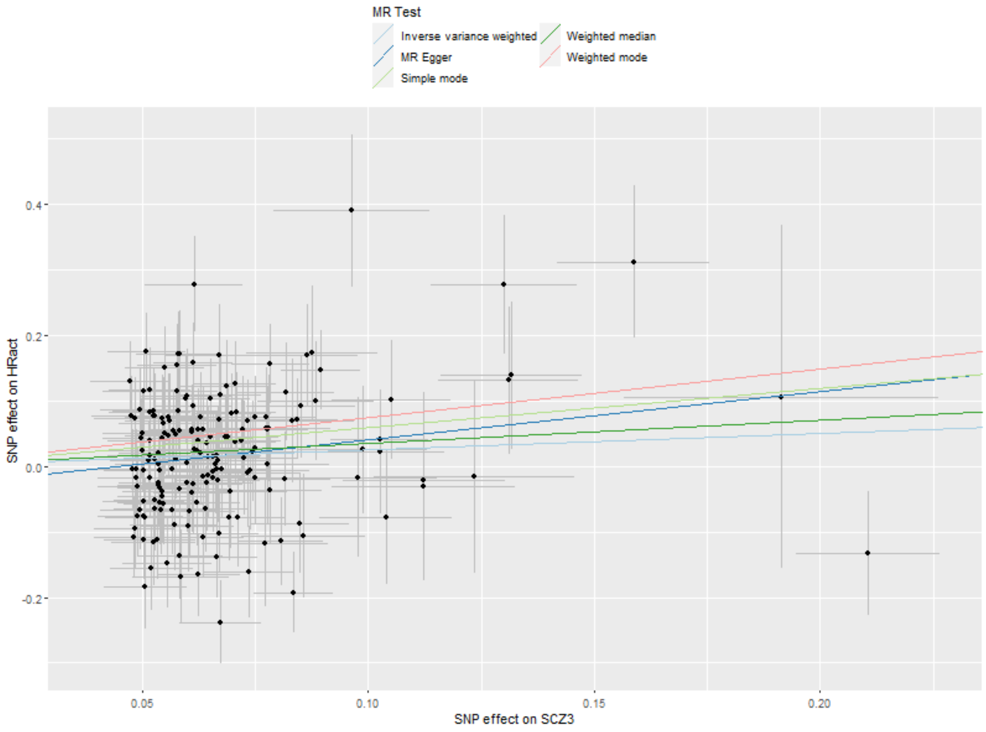

Figure S15. Scatter plot for the Mendelian randomization results with liability to schizophrenia as the exposure and heart rate during activity as the outcome.

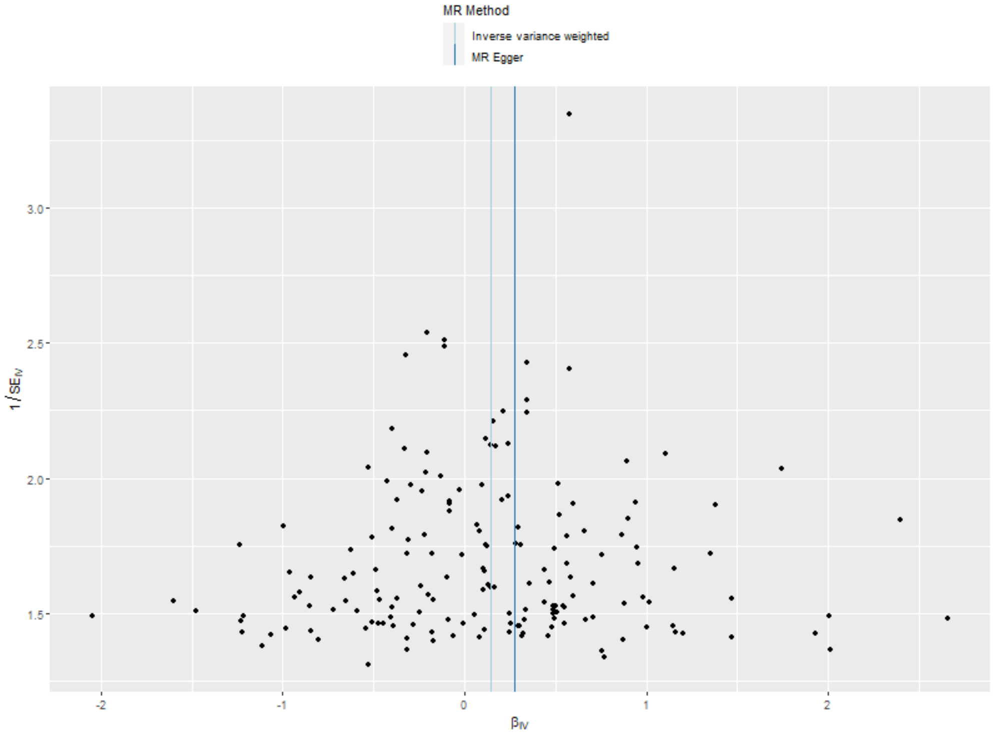

Figure S16. Funnel plot for the Mendelian randomization results with liability to schizophrenia as the exposure and Brugada syndrome as the outcome

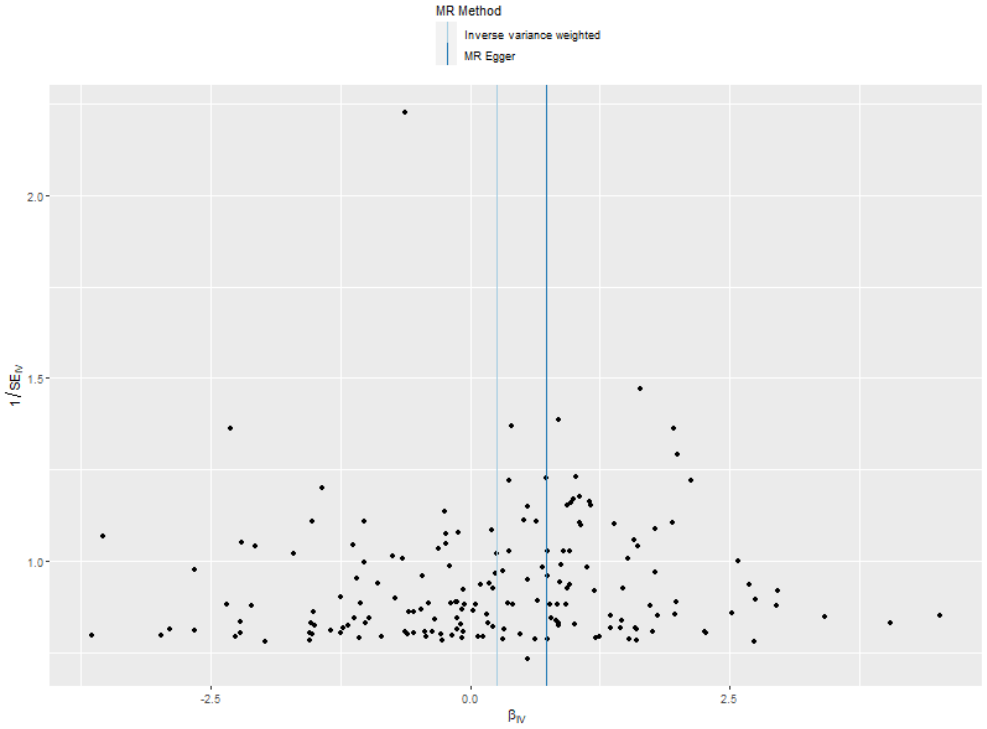

Figure S17. Funnel plot for the Mendelian randomization results with liability to schizophrenia as the exposure and heart rate during activity as the outcome.

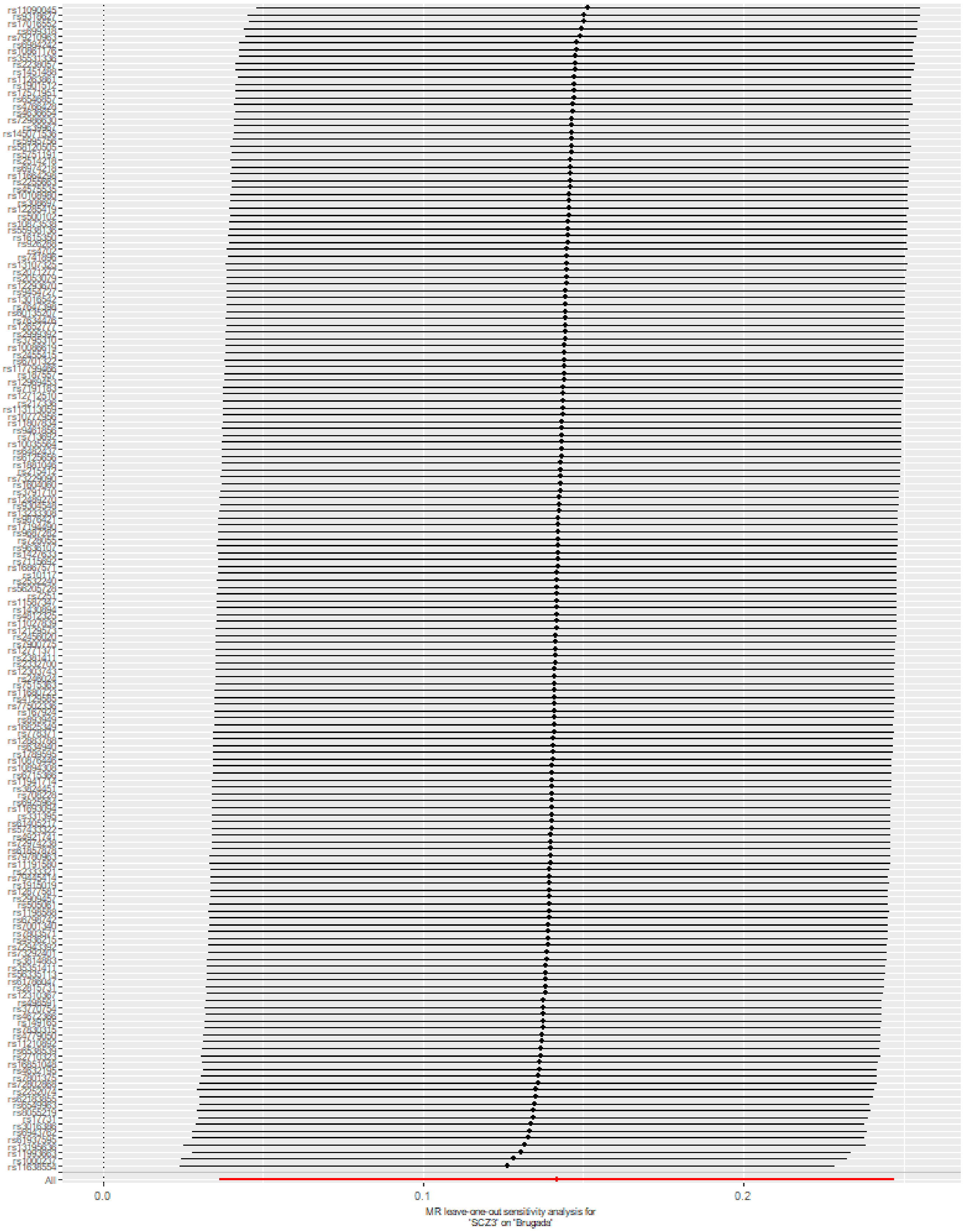

Figure S18. Leave-one-out analysis for the Mendelian randomization results with liability to schizophrenia as the exposure and Brugada syndrome as the outcome.

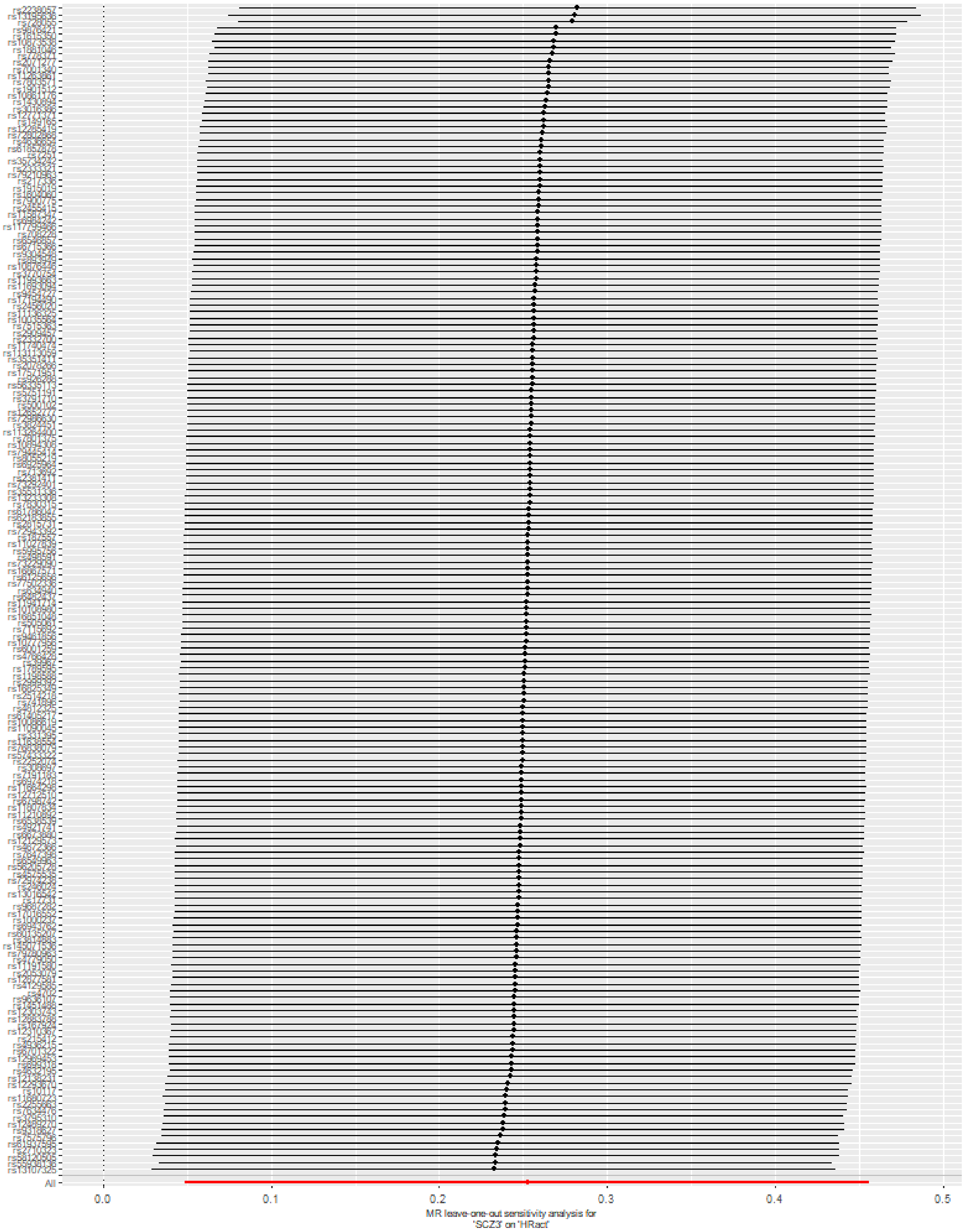

Figure S19. Leave-one-out analysis for the Mendelian randomization results with liability to schizophrenia as the exposure and heart rate during activity as the outcome

Table S3. Test for heterogeneity with Cochran's Q statistic for all univariable MR analyses

Exposure	Outcome	Method	Q	df	P value
Schizophrenia	AF	IVW	280.14	151	8.7E-10
		MR Egger	279.72	150	6.9E-10
Schizophrenia	Brugada	IVW	243.44	168	1.2E-04
		MR Egger	242.94	167	1.3E-04
Schizophrenia	HR activity	IVW	307.00	176	3.6E-09
		MR Egger	304.56	175	4.7E-09
Schizophrenia	HR recovery	IVW	238.03	176	0.001
		MR Egger	237.61	176	0.001
Schizophrenia	HR variability	IVW	118.33	92	0.034
		MR Egger	118.31	91	0.029
Schizophrenia	PR interval	IVW	378.37	172	1.5E-17
		MR Egger	378.31	171	1.5E-17
Schizophrenia	QT interval	IVW	699.96	173	1.7E-64
		MR Egger	698.29	172	1.6E-64
Schizophrenia	JT interval	IVW	596.52	175	2.1E-47
		MR Egger	596.52	174	1.2E-47
Schizophrenia	QRS duration	IVW	438.61	175	1.3E-24
		MR Egger	430.22	174	1.1E-23
AF	Schizophrenia	IVW	172.94	72	2.8E-10
		MR Egger	170.99	71	3.2E-10
Brugada	Schizophrenia	IVW	21.67	11	0.027
		MR Egger	19.44	10	0.035
HR activity	Schizophrenia	IVW	18.34	11	0.074
		MR Egger	18.03	10	0.055
HR recovery	Schizophrenia	IVW	24.88	11	0.009
		MR Egger	20.96	10	0.021
HR variability	Schizophrenia	IVW	11.48	8	0.176
		MR Egger	11.25	7	0.128
PR interval	Schizophrenia	IVW	552.62	253	2.1E-24
		MR Egger	552.62	252	1.4E-24
QT interval	Schizophrenia	IVW	496.88	151	1.8E-38
		MR Egger	495.96	150	1.3E-38
JT interval	Schizophrenia	IVW	309.37	135	1.0E-15
		MR Egger	303.89	134	3.2E-15
QRS duration	Schizophrenia	IVW	253.28	99	1.8E-15
		MR Egger	251.81	98	1.7E-15

Table S4. Multivariable Mendelian randomization

Exposure	Outcome	Additional exposure	IVW<br>beta (95% Cis), p
Schizophrenia	Brugada		0.14 (0.03 to 0.25), 0.009
	Brugada	HR activity	0.19 (-0.02 to 0.40), 0.089
	Brugada	HR recovery	0.17 (-0.04 to 0.38), 0.123
	Brugada	HR variability	0.17 (0.03 to 0.31), 0.015
	Brugada	PR interval	0.13 (-0.04 to 0.30), 0.125
	Brugada	QT interval	0.15 (0.01 to 0.29), 0.042
	Brugada	JT interval	0.16 (0.00 to 0.32), 0.042
	Brugada	QRS duration	0.12 (-0.01 to 0.25), 0.076

Note that the Inverse Variance Weighted (IVW) estimate represents the causal effect of liability to schizophrenia on Brugada, before and after adding each of the additional exposures separately.
